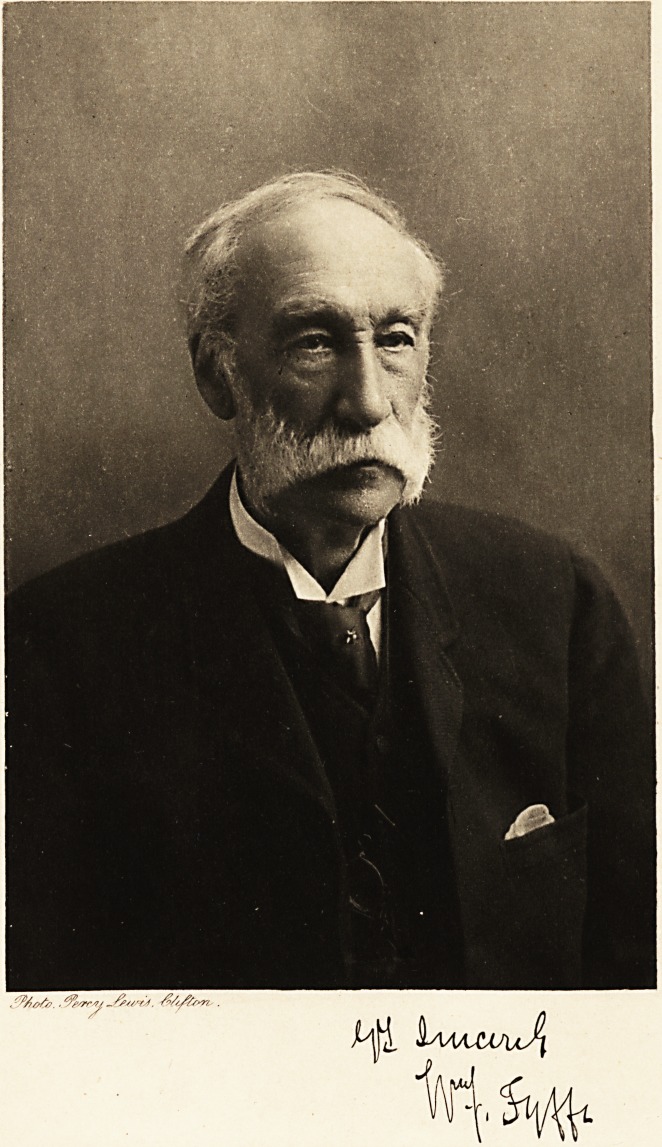# William Johnstone Fyffe

**Published:** 1901-06

**Authors:** 


### WILLIAM JOHNSTONE FYFFE, A.B., M.D.

It is with great regret that we have to record the death of Dr. William Johnstone Fyffe, who passed away at his residence, 2 Rodney Place, Clifton, on May 17th, at the age of 75.

Though of Scotch parentage, he was born in Co. Tyrone and educated in Ireland. He went to Dublin in 1843, entering as a medical student at the Park Street School of Medicine and at the City of Dublin Hospital. In 1847 he took the degrees of A.B. and M.B. in the University of Dublin, and his name was entered on the list of candidates for the Medical Department of the Army at the recommendation of the Marchioness of Abercorn. One year later he was gazetted to the Assistant-Surgeoncy of the 3rd West India Regiment. In 1849 he sailed from Southampton in the *Great Western* steamship for Barbadoes, and from there proceeded to Grenada, where the transport was nearly wrecked. He resided for two years in Jamaica, and when a severe epidemic of Asiatic cholera visited the island, he volunteered to attend the civil population, for which he was presented with an address and a cheque for £250.

After being invalided home, he was for some time at Dover and Fermoy, and on war being declared against Russia in 1854 he was gazetted to the 30th Regiment and ordered to proceed to Bulgaria. He landed in the Crimea on September 14th, and was at the battle of the Alma, and marched to Sebastopol; he was present during part of the siege and at the battle of the 26th of October. He was sent to Balaklava, and afterwards, having contracted fever, was sent for recovery to Scutari Hospital.

In 1855 he came home in medical charge of the transport *Sultana* with 200 wounded and sick, and the passage, which was stormy, lasted 64 days. On his arrival in England the men presented him with a most gratifying address, which was published in the *Times* (March 4th, 1855). He then became one of the Staff of the General Military Hospital, Fort Pitt, Chatham, where nearly all the wounded from the Crimea were collected, and subsequently he was appointed Surgeon to the 13th (Prince Albert's) Light Infantry, and afterwards was gazetted to the Surgeoncy of the 5th Dragoon Guards. In 1863 he was appointed Assistant Professor of Medicine in the Army Medical School at the Royal Victoria Hospital, Netley, and held this position for 10 years.

Having completed 25 years' service in the Army, Dr. Fyffe retired in 1873 with the rank of Deputy Surgeon-General, and came to Clifton. Here he practised as a physician, and in 1876 he unsuccessfully contested election to the Physicianship of the Bristol Royal Infirmary. He became Physician to Clifton College in 1882, and held that post for 15 years, and it is from his connection with Clifton College that he will live long in the memory of his medical *confrères* in Clifton. He was a man especially well suited for the post; his commanding presence, his knowledge of mankind and military discipline, his ripe experience of disease and his quick decision made him an exemplary school physician, and one whom masters, boys, and parents looked up to with profound respect. He took an especial interest in sanitation, and during his tenure of office regularly once a term personally inspected the sanitary condition of all the College houses. On his retiring in 1897 he was presented with a silver bowl from the masters, and many expressions of regret from the Council and all connected with the College.

So great was his popularity in the profession in Bristol that he was the only one unconnected with any public appointment in Bristol who has had the honour of being President of both the local Medical Societies. In 1883 he presided over the Medico-Chirurgical Society, his inaugural address being "On the Nature of the Malarial Poison"; and in 1889 he was President of the Bath and Bristol Branch of the British Medical Association, and delivered a most interesting address on "The Experiences of an Army Surgeon."

In addition to the two presidential addresses, he published a paper on "Diagnosis of Abscess of the Liver," and another on "Epidemic Influenza," introducing an interesting discussion at the Medico-Chirurgical Society in 1890. Other pamphlets which met with great success were "An Address on the Preservation of Health during Schoolboy Life," "A Recent Outbreak of Rötheln" (1892), and "Fifteen Years' Experience of Infectious Diseases in a Public School."

At the Annual Meeting of the British Medical Association which was held in Bristol in 1894, Dr. Fyffe took much interest in the reception, and acted as honorary local treasurer.

After the death of a beloved daughter (Gratian) in 1895, and his wife in 1897, Dr. Fyffe's health began to fail, and his friends saw with dismay that a change was taking place, for instead of being the upright, soldierly man, his gait became more and more stooping, and he gradually grew into the invalid. Medically, his is a somewhat curious case. He consulted Dr. Bright on his return from the Crimea, and came away from his house with the gloomy prognosis of having but few months to live. Since then he had intermittent albuminuria and occasional attacks of gout, but yet lived as a fairly healthy man till his last illness.

He was one of those members of the profession whom we can ill afford to lose. Perhaps somewhat of the old school, his splendid character, his ready sympathy, and his courteous demeanour made him beloved by all who knew him; and if a speech of congratulation, or laudation or condolence, was wanted in private or at a medical meeting, Dr. Fyffe was often the man called upon to make it. At the College, at a club, at the annual medical dinner, and about in Clifton, we shall for a long time greatly lament and feel the loss of our old friend.

The *Journal* Committee have especial reason to feel that they have lost a much valued colleague, on whose aid the Editor could at all times count, as long as his health and strength lasted, for assistance in the reviewing of books and in any other way which the interests of the *Journal* might demand.

We have to thank the family of Dr. Fyffe for the accompanying photogravure, which will be treasured by the subscribers to the *Jounal* as an excellent memento of their old friend.

## Figures and Tables

**Figure f1:**